# Multi-sensory feedback improves spatially compatible sensori-motor responses

**DOI:** 10.1038/s41598-022-24028-5

**Published:** 2022-11-24

**Authors:** A. Dechaux, M. Haytam-Mahsoub, M. Kitazaki, J. Lagarde, G. Ganesh

**Affiliations:** 1 UM-CNRS Laboratoire d’Informatique de Robotique et de Microélectronique de Montpellier (LIRMM), 161, Rue Ada, Montpellier, France; 2 Department of Computer Science and Engineering, Toyohashi University of Technology, Toyohashi, Aichi Japan; 3 Euromov Digital Health in Motion (DHM) Laboratory, University of Montpellier, 700 Avenue du Pic Saint Loup, Montpellier, France

**Keywords:** Sensorimotor processing, Human behaviour

## Abstract

To interact with machines, from computers to cars, we need to monitor multiple sensory stimuli, and respond to them with specific motor actions. It has been shown that our ability to react to a sensory stimulus is dependent on both the stimulus modality, as well as the *spatial compatibility* of the stimulus and the required response. However, the compatibility effects have been examined for sensory modalities individually, and rarely for scenarios requiring individuals to choose from multiple actions. Here, we compared response time of participants when they had to choose one of several spatially distinct, but *compatible,* responses to visual, tactile or simultaneous visual and tactile stimuli. We observed that the presence of both tactile and visual stimuli consistently improved the response time relative to when either stimulus was presented alone. While we did not observe a difference in response times of visual and tactile stimuli, the spatial stimulus localization was observed to be faster for visual stimuli compared to tactile stimuli.

## Introduction

Humans interactions with their environment are modulated by the various stimuli that we receive in multiple sensory modalities. This is particularly so in the case of human machine interfaces which commonly use visual, tactile and auditory stimuli to transmit information to the user^1–4^. An example the parking assistance in a car which uses visual and audio stimuli to indicate the proximity of an obstacle to a particular side of the car. Another example found in the literature is that of a tactile flight envelope “display”, where an airplane flight parameters (which are usually available visually in the cockpit) are fed-back to the pilot using tactile feedback^5^. Previous studies have shown that the speed and accuracy with which we react to a stimulus is not only dependent on the physical property of the stimulus^6–8^ and the nature of the information that is extracted from it^9^, but also on the action that is required as the response, with certain ‘spatially compatible’ stimuli-response couples enabling better performance than other non-compatible ones^10–14^. In this study, we are interested specifically in compatible stimuli-response couples, and in the performance differences induced by the stimulus modality (specifically visual and tactile), when one has to choose from multiple compatible stimuli-response couples.

A 1953 study, by Fitts and Seeger^15^ in which participants had to react to a visual stimulus by moving a stylus towards it, showed that participants reacted faster and more accurately for visual stimuli presented in the same visual hemispace as the responding hand. This effect was termed as “stimulus–response compatibility (SRC)”. This was followed by JR Simon, who showed in 1963^10^ that SRC effects are induced even if the position of the stimulus is irrelevant to the task. Since then, spatial SRC effects have also been exhibited with auditive^13,16^ as well as tactile stimuli^11,16^.

In case of visuo-motor tasks, SRC is determined by the position of the stimulus and response in relation to a *point of reference*^17–20^ in visual space. For example in the Simon Task^10^, it was initially shown that one can respond faster using a hand in the same (compatible) hemispace as the visual stimulus, than with a hand in the opposite (incompatible) hemispace. On the other hand, it is known that for tactile stimuli, compatibility is determined by the relative distribution of stimuli and responses in the somatosensory hemispace^12,17^ (in relation to the body midline). However, these previous studies have used monomodal stimuli^10–17,20^, in which only one sensory modality contains task relevant information, while a secondary sensory modality, if present, is used only as a distractor. Furthermore, these studies traditionally compare the performance between compatible and incompatible stimuli-response couples. Here we are interested in understanding how multimodal stimuli can affect the reaction time of participants in a scenario where the participant has to react to one of multiple possible stimuli and with *only* compatible responses. These *multiple compatible stimulus responses* are typical of many human machine interfaces.

**Figure 1:**
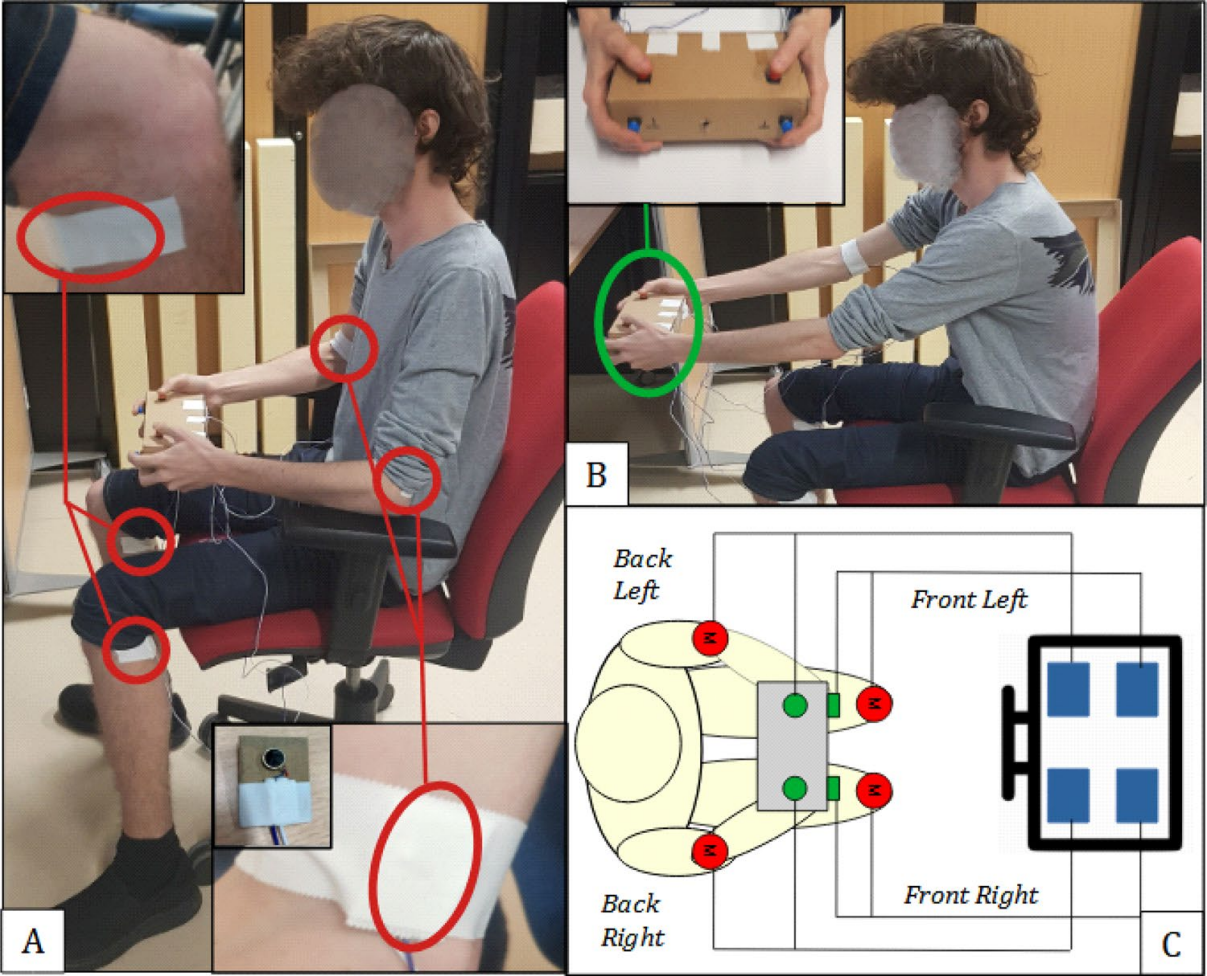
The experimental setup. (**A**) The participant’s sat on a chair in front of a table with a screen. The vibrating motors were located on each knee and elbow of the participant. (**B**) The position of the participant in the Tactile Far condition. In this position the participants held the button box in front of their knees in the anteroposterior axis. (**C**) The mapping between the stimuli (visual or tactile), and the buttons the participants were expected to press corresponding to each stimulus (the screen was placed on the table facing the participant).

Our chosen empirical multiple compatible stimulus response task was motivated by our automobile parking assistance example. The task required participants to react to one of four stimuli representing the *front-left, front-right, back-left* and *back-right* directions on a screen (like in the parking assistance screen of a car) by pressing a *compatible* button, that is a button on the front row and left side (front-left button), front row and right side (front-right button), back row and left side (back-left button) and back row and right side (back-right button) respectively, on a *button box* (see Fig. 1C). The reaction times in this condition were compared with the condition where, instead of the visual stimuli, tactile stimuli were presented near the participant’s two knees (the two front stimuli, given that they sat on a chair) and near their elbows (the two back stimuli). A third condition presented both the tactile and visual stimuli simultaneously.

The act of making actions from perceived cues is often modeled using a drift diffusion model^21,22^ which assumes that a human collects sensory information over time and initiates a decision when his confidence regarding the incoming information passes a given threshold (which determines his reaction time). Given that multi-sensory feedback is known to improve perceptual accuracy^23–25^, it can thus be expected that multi-sensory feedback would also improve the confidence regarding a cue and hence decrease the reaction time for actions for it. We therefore hypothesized that *simultaneous tactile and visual stimuli will reduce the reaction time in our task (Hypothesis 1).*

However, our task requires participants to first perceive and then process the spatial location of the stimuli before activating an appropriate response. In our daily life, we seem to predominantly use visual cues to localize objects and make actions in external space. On the other hand, tactile cues are used predominantly to localize and act in our body space (imagine swatting a mosquito when you feel a pinch on your leg). It is very rare that we need to use tactile cues to decide actions in the external space (like the choice of the buttons on our button box). Due to this dominance of visual cues, and our habituation towards visual cues, we expected *visual stimuli to enable faster stimulus localized response selection compared to tactile stimuli (Hypothesis 2)*.

Finally, during our preliminary experiments, we observed what could be interpreted as a tactile Simon Effect in the finger response along the antero-posterior axis. Specifically, participants were responding faster with some buttons when they held the button box closer to their body in comparison to when they held it near their knee. As a similar effect has never been observed in previous articles, and was contradictory to our intuition as to how the Simon Effect could be expressed in the tactile modality, we decided to include a third hypothesis in the study. We hypothesized that the tactile stimulus–response time will vary with the location of the hand held button box along the antero-posterior axis (Hypothesis 3). However, as we’ll show in the result section, we were unable to reproduce this effect on increasing the participant numbers, suggesting that our observation was a statistical outlier.

## Results

Our participants sat on a chair (see Fig. 1A) in front of a table throughout the experiment. A computer screen presented them with the visual stimuli, while the tactile stimuli were presented using small vibrators fixed near their elbows and knees. They held a *button bo*x with four response buttons, two for the index fingers, and two for the thumbs of their two hands (see inset in Fig. 1B). Figure 1C shows the mapping between each stimulus and the corresponding button the participant was expected to press when he/she experienced the stimulus. Note that the experiment consisted only of compatible couples of stimuli-responses (in terms of SRC), and we did not examine ‘incompatible couples’, in which the stimulus acts as a ‘distractor’ to the response.

All participants took part in six conditions (see methods for details). In three of these conditions they were presented with one stimulus in every trial, at one of the 4 possible locations—a tactile stimulus in the *Tactile condition*, a visual stimulus in the *Visual condition* and a matching pair of tactile and visual stimuli in the *Visuo-Tactile condition*. The button press response time (RT) obtained during these 3 conditions were used to test *Hypothesis 1*, with each condition being repeated 40 times.

The participants also performed 40 trials in a *NOMAP-visual* and *NOMAP-tactile* conditions in which all the visual or all the tactile stimuli were presented simultaneously and which the participant’s could react to a stimulus with any finger. The data obtained in these 2 conditions were compared with the Visual and Tactile conditions to test for *Hypothesis 2*.

Finally, to test for *Hypothesis 3*, the participant performed 40 trials in a *Tactile-Far condition*, which was similar to the Tactile condition except for the location of their response button box While in the Tactile condition the response box was held over the thighs, the box was held beyond the knees in the Tactile-far condition (Fig. 1B).

### Multi-modal stimuli enables faster spatially compatible response times (RTs)

To test our first hypothesis, that visuo-tactile stimuli enables faster compatible response, we examined the averaged RT by the participants across the four stimuli locations in the Visual, Tactile and Visuo-tactile conditions.

**Figure 2:**
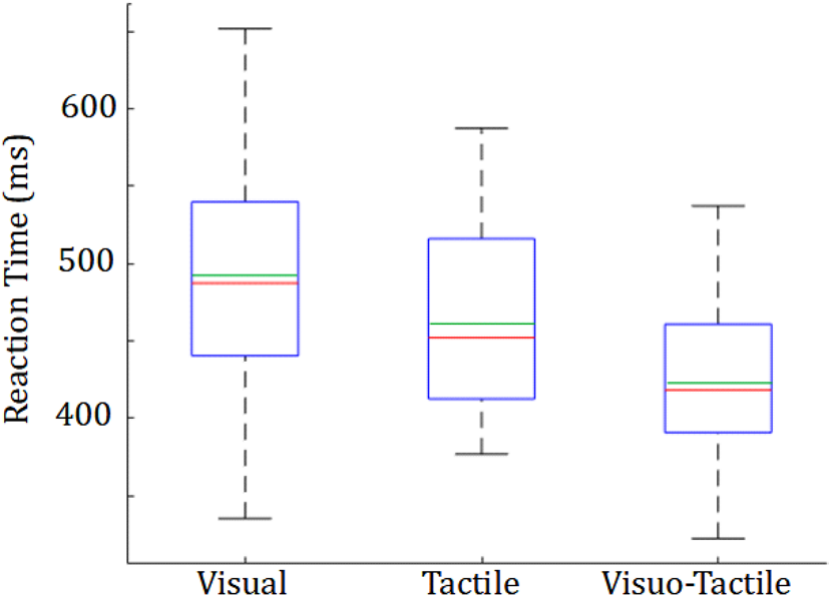
The average reaction time (RT) (averaged across the four directions) presented across participants during the Visual, Tactile and Visuo-Tactile conditions. A clear effect of sensory modality was observed in the results. The median is displayed in red, the mean in green and the first and third quartiles in blue. There were no outliers (non analyzed) data.

Participants exhibited a mean ± standard deviation RT of 489 ± 77sd ms in the Visual condition, 462 ± 60sd ms in the Tactile condition and 420 ± 54sd ms in the Visuo-tactile condition (Fig. 2). A one-way repeated measures ANOVA exhibited a significant effect of the experimental condition on the mean RT [F(2, 46) = 20.62, *p* < 10^–7^, η = 0.17]. A post hoc analysis showed that the participant were responding significantly faster during the Visuo-Tactile condition compared to both the Visual condition [t(23) = 8.05, *p* < 10^–5^, d = 1.01, Bonferroni Corrected] (by 69 ms on average) and the Tactile condition [t(23) = 4.07, *p* < 0.05, d = 0.71, Bonferroni Corrected] (by 27 ms on average). However, no significant difference was observed between the mean RT in the Visual condition compared to the mean RT in the Tactile condition [t(23) = 2.08, *p* > 0.1, d = 0.32 ].

Overall, these results support our first hypothesis that multimodal feedback, in the Visuo-Tactile conditions, reduced RTs in the task. Even though the RTs in the Tactile and Visual conditions are similar in our experiment, these results are constrained by stimulus saliency issues which we discuss in the discussion section.

### Visual stimuli are localized faster than tactile stimuli

However note that the RT in our task consists of three parts, the time corresponding to the detection of a sensory stimulus (modulated by the saliency of the stimuli), the time corresponding to the spatial localization of the stimulus^26^, and the time taken by the motor action. The RT corresponding to the detection and corresponding motor action, are known to be faster with tactile stimuli than with visual stimuli^6,7^ which would suggest that, given that we do not see any differences in our overall RT, the visual stimuli were “localized” faster than the tactile stimuli in our task. To verify this issue (Hypothesis 2) we analyzed RTs in the NOMAP*-*visual and NOMAP*-*tactile conditions respectively.

In the NOMAP conditions, the participants were asked, as in the other conditions, to respond to the stimuli once they perceived it. However, all the four stimuli (visual in case of NOMAP-visual and tactile in case of NOMAP-tactile) were presented simultaneously in these conditions and the participants were allowed to respond to these with any button they chose. We can therefore assume that the RTs in the NOMAP conditions are representative of all the processes in the sensory-motor task except for the ‘stimulus localization’- that is, the process involved in the selection of the spatially correct button based on the stimulus location. Consequently, we can estimate the localization component of the RTs by subtracting the average RTs in the NOMAP-visual and NOMAP-tactile conditions from those in the Visual and Tactile conditions respectively^26,27^.

**Figure 3:**
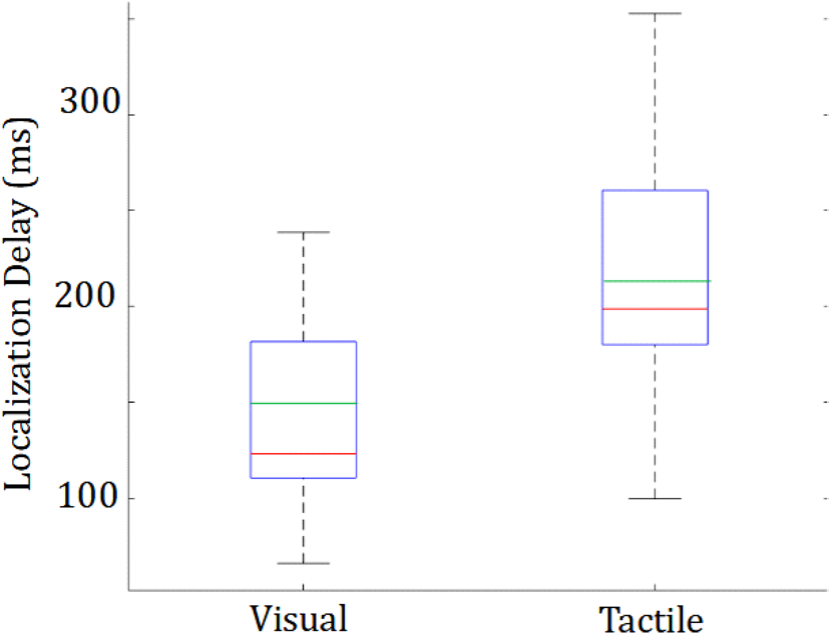
Stimulus spatial localization RT. The difference of the total visual and tactile RT (Fig. 2) relative to the corresponding NOMAP RT provides us with an estimate of the part of the RT representing the stimulus localization time. The median is displayed in red, the mean in green and the first and third quartiles in blue. There were no outliers (non analyzed) data.

The estimate of the visual and tactile localization delay, calculated by the within subject subtraction of their NOMAP-visual and NOMAP-tactile RTs from their Visual and Tactile RTs respectively, are shown in Fig. 3. We observed that the stimulus localization delay was larger (by 69 ms on average) for the tactile (M = 218 ± 61 ms), compared to the visual stimulus (M = 149 ± 61 ms) [t(23) = − 5.77, *p* < 10^–5^, d = 1.13] .

### Response location did not change response time

Finally, we investigated if the average RT to tactile stimulus is dependent on the location of the reaction box.

In which space were the finger responses in our task coded in? One can assume that these responses are coded in the body centric coordinates, that is the same coordinates as the tactile stimulus^12,17^, or in a separate hand centric coordinate, which we here assume to be referenced by the palm. If so then note that in the *Tactile* condition, the participants (held the button box over their thighs) and responded to a stimulus using a button that was *spatially compatible* to the stimulus in both body and hand coordinates. That is, they responded to the front stimuli (near their knees) with their index fingers, which can be considered as the ‘front buttons’ both in body and hand space. And they responded to the back stimuli (near their elbows) with the thumbs, which are ‘back buttons’ both in the hand and body coordinates.

**Figure 4:**
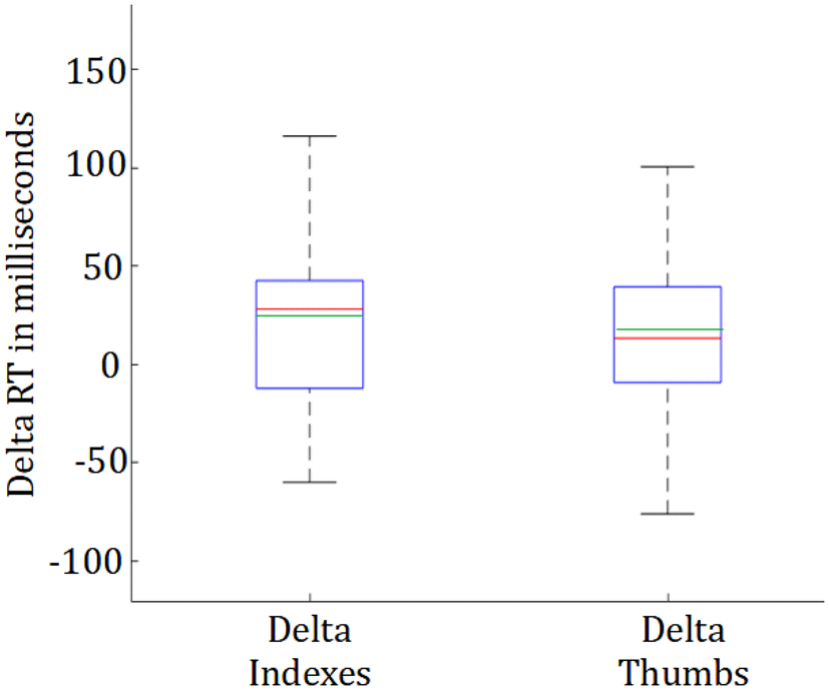
Change in the front and back button RTs between the far- Tactile and Tactile conditions. While we observed a general change in the RT values between the conditions, these changes were not different between the two button sets. The median is displayed in red, the mean in green and the first and third quartiles in blue. There were no outliers (non analyzed) data.

This symmetry is broken in the *Far Tactile* condition, in which the button box was held beyond the knees (see Fig. 1B). In this condition the index and thumb fingers are still the ‘front’ and ‘back’ fingers in the hand centric coordinate (relative to the palm). However, in a body centric coordinate (or the stimulus coordinate), while the front buttons are still in front relative to the front stimuli, the back buttons too are now in front of the front stimuli, potentially affecting the stimulus compatibility. In effect, if the response is coded in the body centric coordinates (Hypothesis 3), we expected the stimulus response compatibility, and hence the RT to change more between the *Tactile* and *Far-Tactile* conditions for the back buttons, than the front buttons. We compare with the front buttons to control for possible comfort related RT changes due to the stretching of the arm in the Far-Tactile condition (Fig. 4).

We observed a slight delay in the reaction time for both front and back tactile RTs between the Tactile and Tactile-far conditions that did not however reach significance ([t(23) = − 1.85, *p* > 0.05, d = 0.33], and [t(23) = − 1.35, *p* > 0.15, d = 0.31] respectively). Crucially, this delay introduced by the Tactile-Far condition did not significantly vary (8 ms on average) between the front (Mean = 25 ± 62 ms) and back (Mean = 17 ± 67 ms) tactile RTs [t(23) = 0.59, *p* > 0.5, d = 0.07] (Fig. 4). Overall, while there was a general change in the response time with the change in the location of the response box, probably due to discomfort related reasons, our results could not confirm changes in stimulus response compatibility between the two conditions, and did not support our Hypothesis 3.

## Discussion

In this study we analyzed specifically a scenario with multiple compatible stimulus–response pairs, and investigated how the localization of the sensory stimuli in this scenario is different with visual or tactile stimuli. The empirical task we chose was motivated by the parking assist system common in modern automobiles, though with visuo-tactile rather than audio-visual cues.

It is well known that tactile-motor responses are faster than visuo-motor responses^6,7^. However, these reports are from tasks in which there was no action choice and participants responded to a single available action. We a priori hypothesized that tactile stimuli will also lead to faster response in our multiple stimuli-responses task. However, we observed that this was not so in our task. We did observe response times (RTs) to be clearly faster in the NOMAP-tactile condition (230 ± 30 ms), compared to the NOMAP-visual condition (340 ± 60 ms) by 110 ms on average. However, spatial localization of the stimulus was observed to be significantly faster with visual stimuli (Fig. 3), eventually leading to no differences in the overall response in the task (Fig. 2).

Several factors may have contributed to the faster spatial localization of visual stimuli. First, visual interfaces, where we need to respond to visual stimuli, are much more common in our daily lives compared to tactile interfaces. Participants are therefore probably more used to spatially localizing visual stimuli. This possibility is also supported by the fact that participants made more errors (double presses) during the *Tactile* condition (1.8 ± 1.8 double presses) in comparison to the *Visual* condition (1.5 ± 1.3 double presses). Second, it is possible that the observations here are modulated by the nature of the stimulation pattern. We used visual and tactile stimuli that were ‘square waves’ in time. That is, when a stimulus was presented, it (color value or vibration intensity) changed from zero to a predetermined constant value and stayed at this level until the participant answered correctly, before dropping back to zero. Other stimulation patterns, especially in case of tactile stimulations, may yield different results. And third, the saliency of signals. Partially again due to habituation, we are probably more sensitive to visual stimuli. The sensitivity of tactile signals on the other hand, is probably lower and dependent on the location on the body where it is presented^17,28^. We tried to maximize the saliency of tactile stimuli by operating the vibratory motors at maximum intensity, and using vibrator phone motors that most participants are expected to be habituated to. However, having said that, we recognize that we cannot ensure normalization of the intensity and saliency of stimuli, in the visual and tactile conditions, as these are two different modalities. To analyze for possible effects of saliency, in fact our visual condition included three groups of participants who worked with visual stimuli of different luminosity (see methods for details). We however could not find any difference between the RTs between the three groups [F(5,10) = 0.96, *p* > 0.4, η = 0.14] and hence combine their RTs in Figs. 2 and 3. However further studies are required to verify how relative intensity between the visual and tactile stimulations can change the results we obtain here.

During preliminary tests of the setup, we observed that the RT changed depending on the position of the button box relative to the body along the antero-posterior axis which was contradictory with our knowledge/intuition of the Simon Effect in the tactile modality. We therefore formulated Hypothesis 3 and added the Far Tactile condition to investigate this issue further. After full data collection however, we were unable to reproduce the effect. This result suggests a hand-centric representation for the action (button press) that seems independent from the frame used to represent tactile stimuli. However, further studies are required to clarify this issue.

Finally, our results should however be understood in light of the population that participated in the study. All our participants were between 18 and 45 years of age and none of them reported a major sight deficiency or tactile insensibility. Subjects in a different age range or impaired in one or the other sensory modality may yield different results. Similarly, participants who have specific training in visuo-motor reactions, like professional athletes and pilots, may exhibit different results than what we observe here. However, it is important to note that having multimodal feedback of both visual and tactile stimuli is clearly beneficial for the general population when one has to respond to one of multiple (compatible) sensory stimuli. Previous studies have shown that humans can improve perception by integrating information from multiple sensory modalities^23–25^. Here we show the same but in regard to reaction time and in a task where an individual has to respond by spatially localizing the stimuli. This result is significant for human–machine interface design, especially in scenarios requiring highly precise and fast responses like when driving an automobile or piloting an airplane.

## Methods

### Participants

24 participants (17 males, 7 females, aged 19–45, mean 26) took part in our study. 20 of them reported being right handed, 4 reported being left handed. All experiments were conducted according to the principles in the Declaration of Helsinki. The subjects gave informed consent prior to the experiment and the experiments were approved by the Comité d’Ethique de Euromov (now merged with the Comité d’Ethique de l’Université de Montpellier) (IRB-UM, Approval ID: 2201F).

Prior to the data collection, a preliminary study with the setup allowed us to estimate an effect size of about 0.35 (η = 0.17) for the ANOVA between the Tactile, Visual and Visuo-Tactile condition. Based on this estimated effect size, we calculated the necessary number of participants as 15 to reach a significance level (α = 0.05) with the G-Power^Ⓡ^ software.

### Setup

The participant sat on a chair in front of a computer screen on a table. The task required participants to react to one of four stimuli representing the *front-left, front right, back-left* and *back-right* directions. The visual stimuli corresponding to these directions were presented as four color-changing squares displayed on a 32inch screen placed 50 cm away from the participant. These squares, initially blue, turn red to represent stimulation. Note that on our computer screen (as in a car*)* the *front* visual stimuli were in fact represented by the top stimuli, while the back stimuli were represented by the bottom stimuli, such that the participants in fact respond with the top stimuli with front buttons and bottom stimuli with back buttons. This has been shown to still be compatible in regards to the visual Simon Effect, which does not discriminate between depth and height^29^. The tactile stimuli were presented using 4 vibrating motors (VC0825B002F) fixated on the user skin in the interior of each knee and elbow and vibrating at a frequency of 225 Hz when powered.

The responses were made by pressing one of 4 buttons distributed on the top and front side of the button box (see Fig. 1). During the experiment, the participant held the device such that their thumbs were resting on the top buttons while their indexes were resting on the front buttons respectively.

Each *trial* started with a random delay chosen between 1250 and 2000 ms before the stimulus onset. The stimulus remained active until the participant pressed the correct button corresponding to the stimulus. When he did, the stimulus and the timer were stopped and his reaction time was recorded as the time between the presentation of stimulus and the button press.

If the participant did not press the correct button within 3500 ms of the trial start, the trial was considered a failure and removed from data analysis.

### Conditions

The experiment was divided into 6 blocks, each corresponding to one of 5 experimental conditions. To avoid the handedness of the participant having an effect on the result, we made sure that every condition contained an equal amount of left/right handed button presses.

The participant started and ended the experiment (block 1 and block 6) with 20 *trials* in the NOMAP condition. This block was further divided in 2 periods of 10 *trials* corresponding to tactile and visual stimuli respectively (*NOMAP Tactile*, *NOMAP Visual*). During this condition, the four stimuli (visual or tactile) were always presented together, and to respond, the participant had to use a specific hand (half of the trial with the left and the other half with their right hand) but were free to choose the responding finger.

The other four blocks (block 2 to 5) of the experiment contained 40 *trials* each and were performed in either the Tactile, Visual, Visuo-Tactile or Far Tactil*e* condition. The order of the conditions was randomized across participants.

A trial in the Tactile, Visual and Visuo-Tactile conditions corresponded to the presentation of one stimuli, either a tactile stimulus on (in the Tactile conditions), a visual stimulus (Visual conditions), or a matching pair of visual and tactile (associated to the same button) turning on simultaneously (in the Visuo-Tactile condition).

The *Far Tactile* condition was the same as the *Tactile* condition with the exception that the participant was asked to hold the response button box beyond the knees, such that all the buttons were further than the knees along the antero-posterior axis (see inset in Fig. 1).

We initially planned to collect data from 15 participants, as suggested by our preliminary effect size analysis. However, after starting the data collection we recognized that the lighting condition in which the study was performed could have an effect on the result (in regards to the saliency of the visual stimuli). To identify if this was the case, we decided to add 9 more participants to analyze the possible effect of luminosity. The participants were divided into three groups corresponding to different screen luminosity: Low (6 participants), Medium (12 participants), High (6 participants). The visual stimuli of the Low group presented a mean luminosity (gray-level) of L = 0.07 and Weber contrast (computed between the stimulus and background) of W = 0.35, for the Medium group of L = 0.24 and W = 0.34 and for the High group of L = 0.38 and W = 0.31. The tactile stimulation was maintained the same for all the groups.

We did not find an effect of this factor [F(2, 10) = 1.06, *p* > 0.35, η = 0.14] and hence combined the data and decided not to analyze this further.

Importantly, we note that we observed an effect size for the ANOVA analysis that was over our initial estimate used to decide the participant numbers, both over the first 15 participants (0.45) as well as overall 24 participants (0.41).

### Data processing

We collected the reaction time (time between stimulus presentation and response button press), or RT, for each trial in every condition. We rejected all the trials in which the participant responded before 150 ms and after 1000 ms of stimulus onset. We also rejected the trials where the participant pressed the wrong button before correcting him/herself. The number of trials rejected was below 2% on average across all participants and conditions and less than 5% across all participants for each condition. In details, rejected trials represented 0.8% (11) of the trial in the NOMAP condition, 4.6% (44) of the trial in the Tactile condition, 3.4% (33) of the trial in the Visual condition, 4.2% (42) of the trial in the Visuo-Tactile condition and 5% (47) of the trial in the Far-Tactile condition. The accepted RTs were averaged across the trials performed in each condition.

First we conducted a one-way Anova on the RT between the three luminosity groups in the Visual condition. We observed that there were no differences between the groups for either the average RT [F(5,10) = 0.96, *p* > 0.4, η = 0.14] or the in the localization time [F(5, 10) = 1.06., *p* > 0.35, η = 0.12 ] and hence combined the data and treated them as one group.

We next conducted a one-way repeated measures ANOVA across the Visual, Tactile and Visuo-tactile conditions (Fig. 2) to analyze our Hypothesis 1.

To estimate the stimulus localization time, we subtracted the average RT in the visual and Tactile conditions from the average RT in the NOMAP-visual and NOMAP-tactile conditions for each participant. This data was collected across participants (Fig. 3) and analyzed with a one sample T-test to test for Hypothesis 2.

Finally, a one sample T-test to test between the change in RTs in the Tactile condition and Tactile-far condition between the front and back buttons was used to evaluate Hypothesis 3.

The normality of the datasets used in the ANOVA performed in the present study was confirmed by a series of Kolmogorov-Smirnoff tests performed independently on each group (*p* << 10^–5^).

## Supplementary Information

Supplementary Information.

## Data Availability

The datasets obtained for each participant and analyzed during the current study are available from the corresponding author on reasonable request.
