# Supplementary material for: Multi-sensory feedback improves spatially compatible sensori-motor responses
Source: Sci Rep. 2022 Nov 24;12:20253. doi: 10.1038/s41598-022-24028-5 (PMC9691706; doi:10.1038/s41598-022-24028-5)

Supplementary Materials : *"An analysis of spatially compatible sensori-motor responses: Multimodal facilitation and visual localization advantage"*

Supplementary Material 1 : The response box.

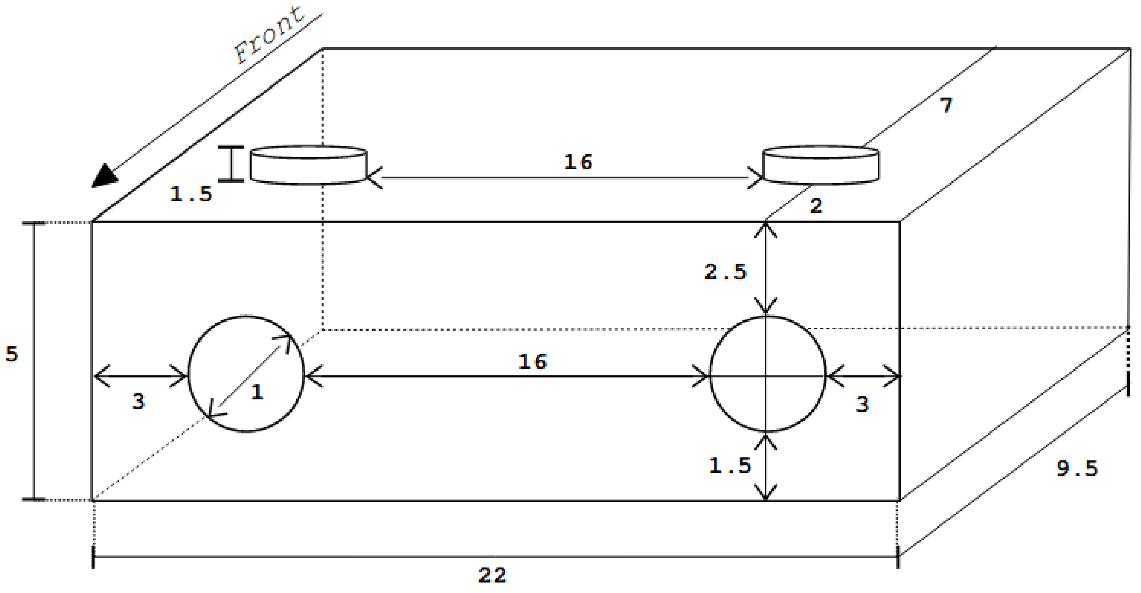

Supplementary Material 2 : The screen (ACER ED270U) and visual stimuli.

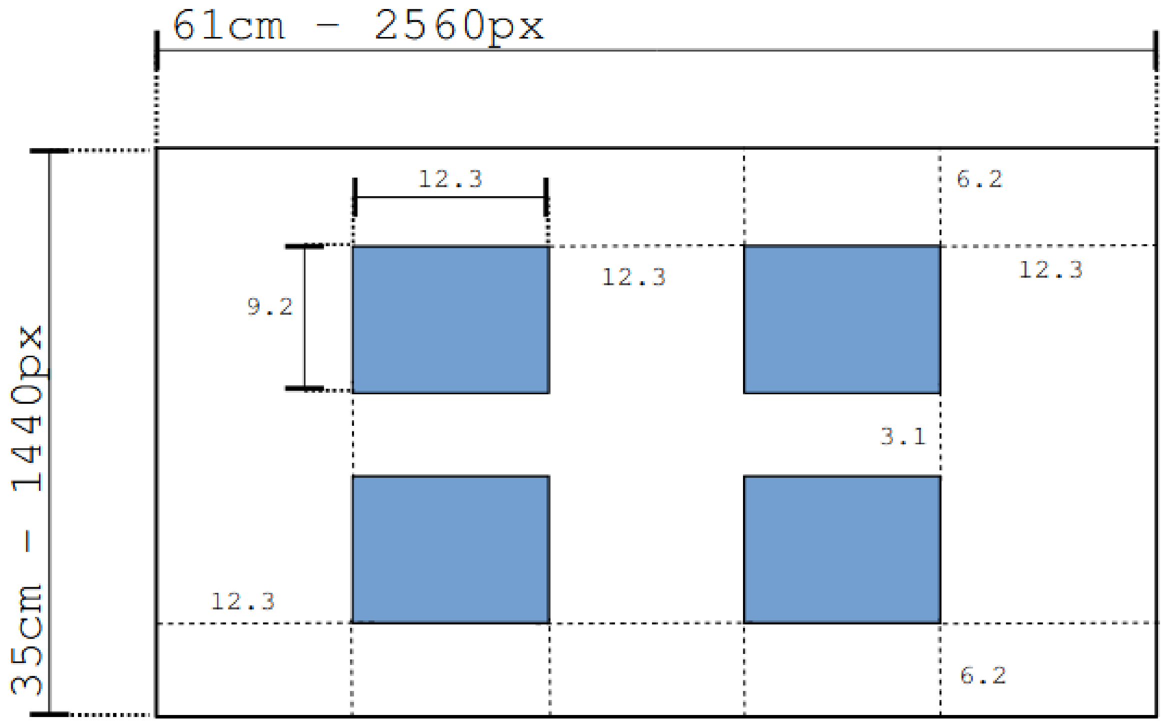

Supplement: Supplementary file 1 — Supplementary Information. [file 41598_2022_24028_MOESM1_ESM.pdf]
